# Hybrid Coronary Revascularisation: Indications, Techniques, and Outcomes

**DOI:** 10.3390/jcm14030880

**Published:** 2025-01-29

**Authors:** Ibrahim T. Fazmin, Jason M. Ali

**Affiliations:** Royal Papworth Hospital, Cambridge CB2 0AY, UK; itf21@cam.ac.uk

**Keywords:** coronary artery disease, coronary artery bypass grafting, percutaneous coronary intervention

## Abstract

Hybrid coronary revascularisation (HCR) integrates coronary artery bypass grafting (CABG) and percutaneous coronary intervention (PCI) to combine the benefits of minimally invasive surgery and advanced stent technology. Typically, HCR involves off-pump left internal mammary artery (LIMA) to left anterior descending artery (LAD) bypass via minimally invasive direct coronary artery bypass (MIDCAB), complemented by PCI to non-LAD vessels. This approach avoids a full sternotomy and cardiopulmonary bypass while preserving the advantages of surgical revascularisation. Patient selection for HCR should be guided by a multidisciplinary heart team, targeting those with severe LAD disease and suitable non-LAD lesions for PCI. This review outlines the surgical techniques, anticoagulation strategies, and procedural sequencing employed in HCR, along with real-world outcomes from observational studies and randomised trials. While current evidence supports the safety and feasibility of HCR in appropriately selected patients, further large-scale randomised trials are needed to clarify its role in comparison to standalone CABG or PCI.

## 1. Introduction

Hybrid coronary revascularisation (HCR) refers to the combination of coronary artery bypass grafting (CABG) and percutaneous coronary intervention (PCI). Commonly, it involves performing a CABG to bypass the left anterior descending artery (LAD) using the left internal mammary artery (LIMA), with PCI performed to other coronary arteries. The procedure can occur concurrently, in what is known as a simultaneous procedure, or as a dual-stage process that involves PCI first then CABG or vice versa. Although minimally invasive CABG via a left anterior thoracotomy was first reported in 1995 [1], the first description of HCR was by Angelini in 1996 [2].

The rationale for HCR is that it possesses the benefits of minimally invasive surgery for the LAD lesion bypass, avoiding a full median sternotomy and leg and/or arm incisions. It can also be performed off pump, avoiding the negative consequences of cardiopulmonary bypass (CPB). Avoiding CPB eliminates the risks of systemic inflammation, coagulopathy and end-organ dysfunction often associated with extracorporeal circulation. This is particularly advantageous in patients with comorbidities such as chronic kidney disease (CKD) or peripheral vascular disease, where the complications of CPB can significantly worsen outcomes. Moreover, the avoidance of sternotomy reduces the risk of wound infections and sternal complications, which are particularly important in patients with diabetes or obesity. A surgical LIMA-LAD anastomosis is the key advantage of this approach, with a large body of evidence for the safety and efficacy of CABG to LAD. In patients with left main disease, the EXCEL trial demonstrated an advantage in terms of reduced need for repeat revascularisation, and a decreased rate of re-infarctions in the LAD territory in CABG vs. PCI [3]. In the SYNTAX study, repeat revascularisation in the PCI arm was mainly due to LAD lesions, and in the CABG arm due to non-LAD lesions [4].

In terms of patient-reported outcomes, there also appears to be a benefit in minimal access CABG versus full-sternotomy CABG. Patients appear to have better quality of life in the months after undergoing an endoscopic CABG [5,6]. A smaller scar will also lead to better cosmesis, as will the avoidance of arm and leg scars from conduit harvesting. The staged nature of dual-stage HCR may also provide psychological benefits, allowing patients time to recover between interventions rather than undergoing a single, highly invasive procedure. However, some patients may experience anxiety related to the interval between MIDCAB and PCI in staged HCR, highlighting the importance of clear communication and shared decision-making in the preoperative period. Thus, HCR combines the advantages of minimally invasive surgery with the benefit of a LIMA to LAD surgical bypass and newer generation drug-eluting stent technology compared to venous conduits.

## 2. Methods

A comprehensive PubMed search was conducted using the keyword “hybrid coronary revascularisation”. The search focused on identifying studies that detailed the techniques employed in HCR, reported outcomes, or provided comparisons of HCR against multivessel CABG and PCI. Peer-reviewed articles were selected for inclusion, particularly those offering insights into patient selection, procedural nuances, and clinical outcomes. The information obtained was synthesised to produce a narrative review, providing a comprehensive overview of the current state of HCR and its role in coronary revascularisation.

## 3. Indications and Patient Selection

The selection of candidates for HCR requires careful consideration of clinical, anatomical, and procedural factors, ideally performed by a multidisciplinary heart team comprising interventional cardiologists and cardiac surgeons. The goal is to identify patients who would benefit most from the complementary strengths of CABG and PCI. The 2018 ESC/EACTS guidelines on myocardial revascularisation give a Class 2b (level of evidence B) recommendation that “hybrid procedures … may be considered in specific patient subsets at experienced centres” [7].

Ideal candidates for HCR will have the following characteristics [8,9]:

- Severe complex lesions of the LAD or left main stem (LMS) which are suitable for CABG with concurrent disease in the circumflex (LCx) or right coronary artery (RCA) territories that are amenable to PCI;
- Low-to-intermediate SYNTAX scores;
- Features predisposing to high risk of wound infection such as peripheral vascular disease and renal failure;
- Characteristics that would make them an otherwise poor surgical candidate such as calcified aorta, history of stroke, significant carotid disease, and lack of suitable conduits;
- Contraindications to multivessel CABG such as intramyocardial non-LAD target vessels.

Conversely, there are also contraindications for HCR [8,9,10]. These include the following:

- Haemodynamic instability or acute shock—due to the time-consuming nature of setting up for MIDCAB and the staged nature of HCR. However, if the haemodynamic instability is due to non-LAD lesions, it may be suitable for PCI-first hybrid HCR with stenting of the culprit lesion followed by MIDCAB.
- Intramyocardial or calcified target sites on the LAD.
- Unsuitability for minimally invasive surgery (e.g., due to previous thoracic surgery, pleural adhesions, or unsuitable anatomy).
- Absence of suitable LIMA for the LAD anastomosis (e.g., due to left subclavian artery stenosis).

For example, consider a 65-year-old male with exertional angina, type 2 diabetes, intermittent claudication, and chronic kidney disease (CKD). If the coronary angiogram reveals a 90% stenosis in the proximal LAD and a 70% stenosis in the mid RCA with a low–intermediate SYNTAX score, the LAD lesion would be ideal for MIDCAB bypass using a LIMA graft, while the RCA lesion is suitable for PCI. Given the patient’s CKD, a MIDCAB ameliorates the risk of sternal dehiscence from a full sternotomy and reduces the risks associated with cardiopulmonary bypass which include renal impairment. A dual-stage HCR approach could be planned, with MIDCAB performed first to address the critical LAD lesion, followed by PCI to the RCA within 48 h. This tailored strategy demonstrates how HCR integrates procedural strengths to address patient-specific risks and anatomical considerations. Tailoring the strategy to individual patient anatomy and comorbidities highlights the versatility and potential of HCR as a modality of revascularisation. Table 1 provides some comparisons between HCR and standalone CABG or PCI, in terms of indications, advantages, and indications.

## 4. Techniques and Procedural Aspects

### 4.1. Minimally Invasive CABG

Minimal-access CABG was first reported in the 1990s. Early publications include a report of an off-pump LIMA to LAD bypass conducted via a left mini-thoracotomy and using thoracoscopy in 1994 [11] and, in 1995, via an anterior mediastinotomy approach with an eight centimetre parasternal incision, also performed on a beating heart [12]. Several variations, configurations, and definitions of minimal-access CABG are employed in current practice, including minimally invasive direct coronary artery bypass (MIDCAB), video-assisted MIDCAB/endoscopic atraumatic coronary artery bypass (EACAB), CABG with cardioplegic arrest and peripheral cardiopulmonary bypass through a mini-thoracotomy, robot-assisted CABG (RA-CABG), and total endoscopic/robot-assisted CABG (TECAB/RA-TECAB) (Table 2) [13,14]. For HCR, however, MIDCAB is the most commonly employed technique for the CABG element of the revascularisation [9].

MIDCAB can be conducted through either lower partial mini-sternotomy (which involves a vertical incision in the midline of the sternum) or left anterior thoracotomy (through the fourth or fifth intercostal space). In our institution, we use both approaches. The left anterior thoracotomy approach also has anaesthetic considerations in that it requires double lumen tube endotracheal intubation to facilitate left lung collapse to facilitate adequate surgical access [15]. A left anterior thoracotomy also has advantages due to being sternum-sparing and avoiding the risk of sternal dehiscence/malunion in comorbid patients. However, it requires a patient who can tolerate single lung ventilation. A lower partial sternotomy is useful in patients with narrow rib spaces and allows for good anatomical exposure and ease of emergency conversion to a full sternotomy.

**Figure 1:**
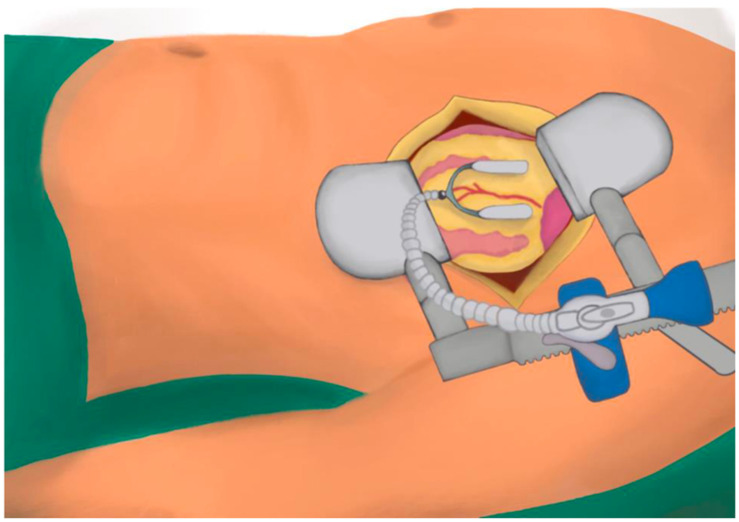
Suction stabiliser used to immobilise the target during a left anterior thoracotomy MIDCAB. Figure used under Open Access Creative Common CC BC license, from an article published by Purmessur et al. [10].

A cardiopulmonary bypass machine on standby is kept within the operating theatre whilst the operation is conducted off-pump, and cell salvage is used [15]. In both approaches, suction stabilisers (Figure 1) (Medtronic Octopus) and intracoronary shunts are used [15].

### 4.2. Sequence and Timing of Procedures

The sequence of procedures can be either both CABG and PCI in the same instance or a dual-stage procedure. There are advantages and disadvantages of using either simultaneous or staged procedures (Table 3), although most centres tend to use a staged approach [9]. Factors determining the order of procedures depend on the patient and presentation of coronary artery disease. The factors favouring CABG first are the same as those favouring a simultaneous HCR procedure [8,9]—namely, the LAD as the culprit lesion for an acute coronary syndrome (ACS) or a critical LAD lesion with stable angina. In our institution, when retrospectively analysed, we performed 66% of staged PCI procedures within 72 h of MIDCAB, with an overall range of 1 to 34 days between MIDCAB and PCI [15].

**Figure 2:**
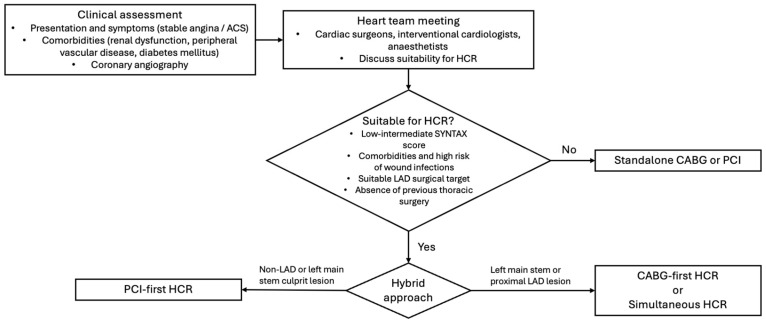
Flowchart for highlighting HCR decision making process. Informed by [8,9,17].

Early PCI following CABG could be undertaken if the non-LAD anatomy has critical lesions. Factors favouring PCI first include ACS due to lesions in non-LAD vessels or critical lesions of non-LAD vessels in stable angina. In these cases, the timing of CABG after PCI can be delayed by 4–6 weeks [8,9]. In one series reported by Sanetra and colleagues, PCI first followed by EACAB in 115 patients had a median time interval of 100 days between stages, with a low rate of major adverse cardiac and cerebrovascular events over a median follow up of 1338 days [16]. A flow chart for conceptualising the decision-making process around HCR is presented in Figure 2.

### 4.3. Anticoagulation and Antiplatelet Strategies

Anticoagulation and antiplatelet agents are required in HCR at the point of LIMA harvesting and deployment of stents to reduce the risk of graft failure or stent thrombosis. Dual antiplatelet therapy (DAPT) confers increased bleeding risk despite its important role in stent protection. Different institutions report different protocols, and there appears to be no consensus in optimal protocols. For example, Willard and colleagues continue DAPT after PCI and during MIDCAB [9]. Their group has shown no difference in bleeding outcomes in a retrospective analysis of patients undergoing MIDCAB with (*n* = 57) and without (*n* = 51) DAPT at the time of surgery [18]. However, another study of 64 patients who were receiving perioperative DAPT, compared to 258 without, demonstrated increased rates of bleeding complications and chest tube drainage in the DAPT group [19]. With regard to DAPT in simultaneous HCR, Kiaii and colleagues described their protocol as follows [17]. They used bivalirudin instead of heparin for the MIDCAB, starting the infusion at the point of LIMA harvest. Then, once haemostasis was achieved, they loaded the patient with clopidogrel or ticagrelor before performing the PCI, overlapping the antiplatelet agent with bivalirudin for one hour before discontinuing the bivalirudin. For two-stage HCR, they used heparin with protamine reversal for MIDCAB then loaded clopidogrel/ticagrelor on the evening of surgery with PCI the next day, continuing the DAPT [17]. Another protocol for simultaneous HCR was described by Sanetra et al. They began a heparin infusion at the time of clipping the LIMA, which is overlapped with ticagrelor given either pre- or intra-operatively [20]. The heparin infusion was not stopped or reversed [20]. An alternative for ACS treated with PCI first with delayed MIDCAB (i.e., non-LAD culprit lesions) was to give DAPT after PCI for four weeks then to perform MIDCAB under single antiplatelet cover [8]. For patients judged to be at risk of bleeding, DAPT can continue for longer and be discontinued immediately prior to surgery or even continued but removed by technologies such as haemo-adsorption during surgery [21,22].

## 5. Outcomes and Real-World Results

### 5.1. Clinical Outcomes

Most of the evidence regarding outcomes of HCR is derived from non-randomised studies, with only a few randomised clinical trials. Additionally, the interpretation of studies for informing treatment choice is made challenging because the comparator, which HCR is held against, could be either multivessel CABG or PCI. Although some centres report an increasing volume of HCR procedures—for example, Dokollari et al. reported an increase in their institution of HCR procedures from 25.5% of all robotic CABG cases in 2005–2010 to 48.4% in 2017–2021 [23]—the overall utilisation of HCR remains low: an analysis of the Society of Thoracic Surgeons database showed in 2011–2013, only 0.48% of total CABG procedures were performed in an HCR approach [17,24].

In the short term, HCR compared to multivessel CABG is associated with superior outcomes in shorter hospital lengths of stay, infection rates, and reduced need for perioperative blood transfusion, with non-inferior outcomes for postoperative acute kidney injury, myocardial infarction, or incidence of atrial fibrillation [8]. However, most of the published outcomes are from non-randomised, retrospective analyses involving propensity matching [8]. In the mid-long term, evidence is sparse but indicates similar outcomes between CABG and HCR in terms of frequency of major adverse cardiac events and mortality, although limited to an average of three years’ of follow up [8].

There have been two randomised control trials (RCTs) comparing CABG with HCR. The HYBRID study randomised patients to either HCR (*n* = 94) or CABG (*n* = 97) and found similar five-year rates of all-cause mortality, myocardial infarction, stroke, and need for repeat revascularisation [25]. The MERGING trial of 40 patients undergoing HCR compared to 20 patients undergoing CABG demonstrated no difference in the primary outcome of all-cause mortality, myocardial infarction, stroke, or need for repeat revascularisation after a two-year follow-up period [26].

There has been no published RCT comparing HCR with PCI. One such study (NCT03089398) was terminated early due to insufficient numbers of patients recruited (200 patients recruited with a target of 2354) [27]. Another study which followed up 200 patients who underwent HCR compared to PCI showed no difference in outcomes [28].

In our institution, we published a retrospective analysis of 82 patients undergoing HCR in a 15-year period compared with 100 patients undergoing isolated MIDCAB [15]. There was no in-hospital or 30-day mortality in the HCR group, although 10-year mortality was worse in the HCR group compared to the MIDCAB-only group (75% vs. 92%, respectively) [15]. However, the HCR group had older patients (64 vs. 61 years of age) and had higher EuroSCORE and worse left ventricular function compared to the MIDCAB group [15]. There were no significant differences between groups in terms of freedom from angina (93% in both groups), freedom from postoperative myocardial infarction (97% in both groups), and freedom from need for repeat revascularisation [15]. When HCR was compared with conventional multivessel CABG by propensity matching with a cohort of CABG patients with similarly revascularized territories, the HCR group had a shorter postoperative length of stays in hospital (5.5 days vs. 7 days) and similar long-term survival [15]. Thus, the outcomes suggest that HCR is a safe option in appropriately chosen patients, and it has satisfactory short- and long-term outcomes.

### 5.2. Financial Implications

Another dimension to consider is one of financial implications, as there may be a balance between procedural expenses and post-procedural benefits. A study by Bachinsky et al. compared 25 patients undergoing HCR with 27 patients who had multivessel off-pump CABG (OPCABG) [29]. While HCR patients experienced fewer postoperative blood transfusions, shorter hospital stays, and better quality-of-life metrics, the higher procedural costs of HCR led to greater overall expenses, perhaps due to equipment requirements and the cost of drug-eluting stents [29]. Halkos et al. examined the contribution margin, which refers to a measure of the difference between hospital reimbursement and total variable costs, and found that HCR had a higher contribution margin compared to OPCABG [5]. This suggests that despite its higher upfront costs, HCR can result in greater financial benefit for hospitals. Furthermore, the HCR group in this study demonstrated reduced transfusion requirements, less time on mechanical ventilation, and shorter postoperative hospital stays, which may translate into lower post-procedural care costs [5]. These findings underscore dual advantages of HCR, enhanced patient outcomes and potentially higher hospital reimbursement, making it a financially viable option in appropriately selected patients.

### 5.3. Learning Curve and Training

When setting up an HCR programme, it is also crucial to consider the learning curve for MIDCAB. Holzhey et al., using cumulative sum analysis, demonstrated that approximately 50–100 MIDCAB cases are required to achieve optimal results in terms of operative time, complication rates, and mortality [30]. This underscores the importance of both individual and institutional experience, as increased procedural frequency is associated with lower failure rates, advocating for HCR to be performed predominantly in high-volume centres. Similarly, Kanber et al. identified a learning curve of around 50 cases for MIDCAB, after which significant improvements in operation time, anastomosis duration, and postoperative length of stay were observed [31]. Additionally, another study highlighted that endoscopic LIMA harvest and total endoscopic CABG procedure times decrease markedly with experience, with the steepest improvement occurring over the first 15–20 cases [32]. These findings emphasize the need for dedicated training and the centralization of HCR at specialized centres to ensure safe and effective implementation.

Several strategies can be used to facilitate wider adoption of HCR. Firstly, concentrating HCR to high-volume centres which have experienced cardiac surgical and interventional cardiology services will allow regular exposure to HCR and help maintain competency. Increased procedural volume (both institutional and individual) is correlated with better outcomes in terms of lower operative mortality and complication rates [33,34]. Training programmes should also be set up which focus on minimal access cardiac surgery techniques, with the inclusion of simulation-based learning (for example, wet lab or cadaveric simulators), which can further augment the learning curve [35]. Proctors who are experienced operators in minimal access cardiac surgery can aid teams who are going through the learning curve whilst also monitoring safety outcomes.

## 6. Conclusions

HCR is purported to offer a “best of both worlds” approach to coronary revascularisation in of multivessel coronary artery disease. Benefits include the minimal access nature of MIDCAB, avoiding cardiopulmonary bypass, long-term prognostic benefit of LIMA to LAD bypass and improved performance of newer generation drug-eluting stents compared to venous conduits. Carefully chosen patients by a heart team stand to benefit from HCR, although evidence is limited and large-scale RCTs have yet to prove the superiority of HCR versus CABG or PCI.

## Figures and Tables

**Table 1:** Comparison of HCR against standalone CABG or PCI.

Aspect	HCR	Standalone CABG	PCI
Invasiveness	Minimally invasive	Highly invasive	Least invasive
Recovery time	Faster recovery than CABG due to small incisions and off-pump surgery	Longer recovery time due to full sternotomy and use of CPB	Fastest recovery–no incisions
Advantages	“Best of both worlds” approach: long term patency of LIMA with least invasive nature of non-LAD revascularisation Avoids CPB and associated sequelae Reduce bleeding, wound complications compared to CABG	Well-established long-term outcomes for multivessel coronary artery disease Can achieve complete revascularisation of all target vessels	Suitable for high-risk surgical candidates Shortest hospital length stay
Limitations	Complex to set up, required hybrid operating theatre and multidisciplinary teams Steep surgical learning curve to perform off pump, minimal access CABG Requires careful anticoagulation and antiplatelet strategies	Higher risks of wound complications and complications from CPB, especially in comorbid patients	Less durable compared to LIMA-LAD bypass compared to CABG or HCR

**Table 2:** Comparison of surgical techniques which can be employed for the surgical aspect of HCR.

Surgical Approach	Description and Application in HCR
MIDCAB	Access through mini-thoracotomy or lower partial mini-sternotomy. Most common surgical technique for HCR.
EACAB (EACAB)	Combines MIDCAB with thoracoscopy to enhance access and precision.
RA-MIDCAB	Robot assistance for improved precision in LIMA harvesting and anastomosis.
TECAB	Fully endoscopic robotic approach. Rarely employed due to complex and time-consuming set-up.

**Table 3:** Comparison of simultaneous versus staged HCR.

	Simultaneous HCR	Staged HCR
Advantages	Complete revascularisation achieved in one stage Potentially decreased overall hospital stay	Can optimise patient between stages Easier management of anticoagulation and antiplatelet agents between procedures
Disadvantages	Higher complexity procedure Challenging to manage anticoagulation and antiplatelets Logistics difficult to organise (requires hybrid operating theatre and attendance of interventional cardiologist and cardiac surgeon)	Prolongs treatment duration and delays complete revascularisation Interval adverse events (e.g., myocardial infarction) can occur

## Data Availability

Data is contained in this article.

## Abbreviations

The following abbreviations are used in this manuscript:

ACS	Acute Coronary Syndrome
CABG	Coronary Artery Bypass Grafting
DAPT	Dual Antiplatelet Therapy
EACTS	European Association for Cardio-Thoracic Surgery
ESC	European Society of Cardiology
HCR	Hybrid Coronary Revascularisation
LAD	Left Anterior Descending Artery
LCx	Left Circumflex Artery
LIMA	Left Internal Mammary Artery
LMS	Left Main Stem
MIDCAB	Minimally Invasive Direct Coronary Artery Bypass
PCI	Percutaneous Coronary Intervention
RA-CABG	Robot-Assisted Coronary Artery Bypass Grafting
RA-TECAB	Robot-Assisted Total Endoscopic Coronary Artery Bypass
RCA	Right Coronary Artery
SYNTAX	Synergy Between PCI With Taxus and Cardiac Surgery
TECAB	Total Endoscopic Coronary Artery Bypass

## References

[1] Calafiore A.M., Angelini G.D. (1996). Left Anterior Small Thoracotomy (LAST) for Coronary Artery Revascularisation. Lancet.

[2] Angelini G., Wilde P., Salerno T., Bosco G., Calafiore A. (1996). Integrated Left Small Thoracotomy and Angioplasty for Multivessel Coronary Artery Revascularisation. Lancet.

[3] Baron S.J., Chinnakondepalli K., Magnuson E.A., Kandzari D.E., Puskas J.D., Ben-Yehuda O., Van Es G.-A., Taggart D.P., Morice M.-C., Lembo N.J. (2017). Quality-of-Life After Everolimus-Eluting Stents or Bypass Surgery for Left-Main Disease: Results From the EXCEL Trial. J. Am. Coll. Cardiol..

[4] Parasca C.A., Head S.J., Milojevic M., Mack M.J., Serruys P.W., Morice M.-C., Mohr F.W., Feldman T.E., Colombo A., Dawkins K.D. (2016). Incidence, Characteristics, Predictors, and Outcomes of Repeat Revascularization After Percutaneous Coronary Intervention and Coronary Artery Bypass Grafting: The SYNTAX Trial at 5 Years. JACC Cardiovasc. Interv..

[5] Halkos M.E., Ford L., Peterson D., Bluestein S.M., Liberman H.A., Kilgo P., Puskas J.D., Guyton R.A., Chowdhury R. (2014). The Impact of Hybrid Coronary Revascularization on Hospital Costs and Reimbursements. Ann. Thorac. Surg..

[6] Bonaros N., Schachner T., Wiedemann D., Oehlinger A., Ruetzler E., Feuchtner G., Kolbitsch C., Velik-Salchner C., Friedrich G., Pachinger O. (2009). Quality of Life Improvement after Robotically Assisted Coronary Artery Bypass Grafting. Cardiology.

[7] Neumann F.-J., Sousa-Uva M., Ahlsson A., Alfonso F., Banning A.P., Benedetto U., Byrne R.A., Collet J.-P., Falk V., Head S.J. (2018). 2018 ESC/EACTS Guidelines on myocardial revascularization. Eur. Heart J..

[8] Thielmann M., Bonaros N., Barbato E., Barili F., Folliguet T., Friedrich G., Gottardi R., Legutko J., Parolari A., Punjabi P. (2024). Hybrid Coronary Revascularization: Position Paper of the European Society of Cardiology Working Group on Cardiovascular Surgery and European Association of Percutaneous Cardiovascular Interventions. Eur. J. Cardio-Thoracic Surg..

[9] Willard R., Scheinerman J., Pupovac S., Patel N.C. (2024). The Current State of Hybrid Coronary Revascularization. Ann. Thorac. Surg..

[10] Purmessur R., Wijesena T., Ali J. (2023). Minimal-Access Coronary Revascularization: Past, Present, and Future. J. Cardiovasc. Dev. Dis..

[11] Benetti F. (1994). Uso de La Toracoscopeia En Cirugia Coronaria Para Diseccion de La Arteria Mamaria Interna. Prensa Med. Argent..

[12] Stanbridge R., Symons G.V., Banwell P.E. (1995). Minimal-Access Surgery for Coronary Artery Revascularisation. Lancet.

[13] Bonatti J., Wallner S., Crailsheim I., Grabenwöger M., Winkler B. (2021). Minimally invasive and robotic coronary artery bypass graftinga 25-year review. J. Thorac. Dis..

[14] Vassiliades T.A. (2003). Endoscopic-Assisted Atraumatic Coronary Artery Bypass. Asian Cardiovasc. Thorac. Ann..

[15] Farid S., Ali J.M., Stohlner V., Alam R., Schofield P., Nashef S., De Silva R. (2018). Long-Term Outcome of Patients Undergoing Minimally Invasive Direct Coronary Artery Bypass Surgery: A Single-Center Experience. Innov. Technol. Tech. Cardiothorac. Vasc. Surg..

[16] Sanetra K., Buszman P.P., Jankowska-Sanetra J., Konopko M., Slabon-Turska M., Białek K., Milewski K., Gerber W., Bochenek A., Kachel M. (2023). Safety and Feasibility of Minimally Invasive Coronary Artery Bypass Surgery Early after Drug-Eluting Stent Implantation Due to Acute Coronary Syndrome. Polish Heart J. Kardiol. Polska.

[17] Kiaii B., Teefy P. (2019). Hybrid Coronary Artery Revascularization: A Review and Current Evidence. Innov. Technol. Tech. Cardiothorac. Vasc. Surg..

[18] Hemli J.M., Darla L.S., Panetta C.R., Jennings J., Subramanian V.A., Patel N.C. (2012). Does Dual Antiplatelet Therapy Affect Blood Loss and Transfusion Requirements in Robotic-Assisted Coronary Artery Surgery? Innovations(Phila). Innov. Technol. Tech. Cardiothorac. Vasc. Surg..

[19] Daniel W.T., Liberman H.A., Kilgo P., Puskas J.D., Vassiliades T.A., Devireddy C., Jaber W., Guyton R.A., Halkos M.E. (2014). The Impact of Clopidogrel Therapy on Postoperative Bleeding after Robotic-Assisted Coronary Artery Bypass Surgery. Eur. J. Cardio-Thoracic Surg..

[20] Sanetra K., Buszman P.P., Jankowska-Sanetra J., Cisowski M., Fil W., Gorycki B., Bochenek A., Slabon-Turska M., Konopko M., Kaźmierczak P. (2022). One-Stage Hybrid Coronary Revascularization for the Treatment of Multivessel Coronary Artery Disease—Periprocedural and Long-Term Results from the “HYBRID-COR” Feasibility Study. Front. Cardiovasc. Med..

[21] Amabile N., Chiarito M., Lee V.T., Angiolillo D.J., Capodanno D., Bhatt D.L., Mack M.J., Storey R.F., Schmoeckel M., Gibson C.M. (2023). Reversal and Removal of Oral Antithrombotic Drugs in Patients with Active or Perceived Imminent Bleeding. Eur. Heart J..

[22] Tripathi R., Morales J., Lee V., Gibson C.M., Mack M.J., Schneider D.J., Douketis J., Sellke F.W., Ohman M.E., Thourani V.H. (2022). Antithrombotic Drug Removal from Whole Blood Using Haemoadsorption with a Porous Polymer Bead Sorbent. Eur. Heart J. Cardiovasc. Pharmacother..

[23] Dokollari A., Sicouri S., Erten O., Gray W.A., Shapiro T.A., McGeehin F., Badri M., Coady P., Gnall E., Caroline M. (2024). Long-Term Clinical Outcomes of Robotic-Assisted Surgical Coronary Artery Revascularisation. EuroIntervention.

[24] Harskamp R.E., Bagai A., Halkos M.E., Rao S.V., Bachinsky W.B., Patel M.R., De Winter R.J., Peterson E.D., Alexander J.H., Lopes R.D. (2014). Clinical Outcomes after Hybrid Coronary Revascularization versus Coronary Artery Bypass Surgery: A Meta-Analysis of 1,190 Patients. Am. Heart J..

[25] Tajstra M., Hrapkowicz T., Hawranek M., Filipiak K., Gierlotka M., Zembala M., Gąsior M., Zembala M.O. (2018). POL-MIDES Study Investigators Hybrid Coronary Revascularization in Selected Patients With Multivessel Disease: 5-Year Clinical Outcomes of the Prospective Randomized Pilot Study. JACC Cardiovasc. Interv..

[26] Esteves V., Oliveira M.A.P., Feitosa F.S., Mariani J., Campos C.M., Hajjar L.A., Lisboa L.A., Jatene F.B., Filho R.K., Neto P.A.L. (2020). Late Clinical Outcomes of Myocardial Hybrid Revascularization versus Coronary Artery Bypass Grafting for Complex Triple-Vessel Disease: Long-Term Follow-up of the Randomized MERGING Clinical Trial. Catheter. Cardiovasc. Interv..

[27] Gaudino M., Sandner S. (2024). Hybrid Coronary Revascularisation: The Best or the Worst of Both Worlds?. EuroIntervention.

[28] Puskas J.D., Halkos M.E., DeRose J.J., Bagiella E., Miller M.A., Overbey J., Bonatti J., Srinivas V.S., Vesely M., Sutter F. (2016). Hybrid Coronary Revascularization for the Treatment of Multivessel Coronary Artery Disease: A Multicenter Observational Study. J. Am. Coll. Cardiol..

[29] Bachinsky W.B., Abdelsalam M., Boga G., Kiljanek L., Mumtaz M., Mccarty C. (2012). Comparative Study of Same Sitting Hybrid Coronary Artery Revascularization versus Off-Pump Coronary Artery Bypass in Multivessel Coronary Artery Disease. J. Interv. Cardiol..

[30] Holzhey D.M., Jacobs S., Walther T., Mochalski M., Mohr F.W., Falk V. (2007). Cumulative Sum Failure Analysis for Eight Surgeons Performing Minimally Invasive Direct Coronary Artery Bypass. J. Thorac. Cardiovasc. Surg..

[31] Kanber E.M., Köseoğlu M., Şahin M. (2023). Assessing the Learning Curve of the Minimally Invasive Direct Coronary Artery By-Pass Technique. Eur. Arch. Med Res..

[32] Akca F., Woorst J.T. (2023). Learning Curve of Thoracoscopic Nonrobotic Harvest of the Left Internal Mammary Artery in Minimally Invasive Coronary Artery Bypass Grafting. Innov. Technol. Tech. Cardiothorac. Vasc. Surg..

[33] Birkmeyer J.D., Siewers A.E., Finlayson E.V.A. (2002). Hospital Volume and Surgical Mortality in the United States. N. Engl. J. Med..

[34] Birkmeyer J.D., Stukel T.A., Siewers A.E., Goodney P.P., Wennberg D.E., Lucas F.L. (2003). Surgeon Volume and Operative Mortality in the United States. N. Engl. J. Med..

[35] Rad A.A., Hajzamani D., Nia P.S. (2023). Simulation-Based Training in Cardiac Surgery: A Systematic Review. Interdiscip. Cardiovasc. Thorac. Surg..

